# Sulfonated Pentablock Copolymer Coating of Polypropylene Filters for Dye and Metal Ions Effective Removal by Integrated Adsorption and Filtration Process

**DOI:** 10.3390/ijms231911777

**Published:** 2022-10-04

**Authors:** Simona Filice, Viviana Scuderi, Sebania Libertino, Massimo Zimbone, Clelia Galati, Natalia Spinella, Leon Gradon, Luciano Falqui, Silvia Scalese

**Affiliations:** 1 Consiglio Nazionale delle Ricerche, Istituto per la Microelettronica e Microsistemi (CNR-IMM), Ottava Strada n.5, 95121 Catania, Italy; 2 Consiglio Nazionale delle Ricerche, Istituto per la Microelettronica e Microsistemi (CNR-IMM), Via S. Sofia 64, 95123 Catania, Italy; 3 STMicroelectronics Stradale Primosole 50, 95121 Catania, Italy; 4 Faculty of Chemical and Process Engineering, Warsaw University of Technology, ul. Warynskiego 1, 00-645 Warsaw, Poland; 5 Plastica Alfa SpA, Zona Industriale, C.da S.M.Poggiarelli, 95041 Caltagirone (CT), Italy

**Keywords:** sulfonated pentablock copolymer, ions removal, adsorption, filtration

## Abstract

In this work, we coated polypropylene (PP) fibrous filters with sulfonated pentablock copolymer (s-PBC) layers and tested them for the removal of cationic organic dyes, such as methylene blue (MB), and heavy metal ions (Fe^3+^ and Co^2+^) from water by adsorption and filtration experiments. Some of the coated filters were irradiated by UV light before being exposed to contaminated water and then were tested with unirradiated filters in the same adsorption and filtration experiments. Polymer-coated filters showed high efficiency in removing MB from an aqueous solution in both absorption and filtration processes, with 90% and 80% removal, respectively. On the other hand, for heavy metal ions (Fe^3+^ and Co^2+^), the coated filters showed a better removal performance in the filtration process than for the adsorption one. In fact, in the adsorption process, controlled interaction times allow the ionic species to interact with the surface of the filters leading to the formation and release of new species in solution. During filtration, the ionic species are easily trapped in the filters, in particular by UV modified filters, and we observed for Fe^3+^ ions a total removal (>99%) in a single filtration process and for Co^2+^ ions a larger removal with respect to the untreated filter. The mechanisms involved in the removal of the contaminants processes were investigated by characterizing the filters before and after use by means of scanning electron microscopy (SEM) combined with energy-dispersive X-ray (EDX) analysis and Fourier transform infrared spectroscopy (FT-IR).

## 1. Introduction

Nowadays, a large part of the world’s inhabitants has limited access to clean water, and this is one of the most dramatic effects deriving from anthropogenic activities. This problem is expected to increase due to the high rate of world population growth and, in the coming years, it will also affect regions currently considered to be rich in water. Harmful and toxic waste produced by industrial and agricultural activities ends up in the waterbodies and, therefore, new effective strategies are required to prevent pollution or to remove it from water.

For this reason, much effort is dedicated to find new remediation methods to purify water, efficiently, with less energy, minimizing the consumption of chemical compounds. Much attention is also given to the impact that the various techniques/materials used have on the environment.

Research is mainly focused on four fronts: disinfection, decontamination, water reuse and desalination [1].

Several conventional techniques were used to reduce toxic dye compounds in wastewater, including separation [2], reverse osmosis, precipitation, ion exchange method, and ultra-filtration adsorption on activated carbon [3]. The greater disadvantage of these techniques is the formation of secondary waste that cannot be reprocessed.

Instead, photocatalytic methods consist of highly advanced oxidation processes that are used for the photodegradation of toxic compounds, showing high efficiency, simple operation, low cost, and low energy consumption [4]. Many photocatalytic materials are known, including ZnO [5], TiO_2_ [6], BiOBr [7], and WO_3_ [8].

In this context, membrane technology plays an important role in water treatment due to its interesting features, easy operation, no addition of chemical additives (or less), cost-efficiency, high productivity, easy scaling, and suitable removal capacity. A membrane is a selective system that allows wanted materials to pass through and unwanted to be retained on the membrane surface [9].

The current membranes are based on some conventional polymeric materials, and they frequently suffer from limited chemical, mechanical and thermal stabilities [10]. Inorganic membranes that can achieve high selectivity and/or high permeability are also weak, expensive and difficult to expand [11]. In this context, the development of innovative new generation membrane material, such as two-dimensional (2D) porous organic polymers (POPs), is receiving great attention due to highly tunable pores/channels, robust frameworks/networks, intrinsic flexibility, and light weight for multiple separation purposes. It has recently been shown that the filtering properties of a membrane, as well as the resistance to acidic environments, can be increased either by modifying the surface of the polymer or by incorporating polymers/molecules with different structures and properties [12,13,14].

In our previous works [15,16,17,18], we showed that sulfonilic groups of sulfonated pentablock copolymer (s-PBC) are highly hydrophilic, negatively charged, and acidic, making this polymer a suitable high capacitive adsorbent for the removal of cationic species. In particular, membranes and s-PBC/graphene oxide (GO) nanocomposite membranes show suitable adsorption abilities for the removal of different heavy metal ions (Ni^2+^, Co^2+^, Cr^3+^, and Pb^2+^) from aqueous solutions containing the corresponding metal salts at different concentrations, due to the presence of sulfonic groups that play a fundamental role in the adsorption process of metal ions. Starting from these considerations, we proposed for the first time the application of s-PBC (Nexar^TM^) as a multifunctional coating for water filters [19,20]: due to hydrophilicity, acidity, and smoothness of the coating layer, the adhesion, and proliferation of planktonic *P. aeruginosa* was contrasted by a combined repulsion and contact killing mechanism avoiding the biofilm formation. This polyvalent antimicrobial nature of Nexar^TM^ could provide a substantial improvement to the surface coatings for multi-purpose filters with antifouling properties and simultaneous removal of bacteria and cationic contaminants from water. Furthermore, this strategy allows reducing the use of chemical and toxic agents to avoid the biofouling effect, increasing the filter’s lifespan.

To take advantage of the described properties, in this work polypropylene (PP) fibrous filters were coated by a s-PBC layer (s-PBC@PP) and tested in adsorption and filtration processes for a fast and efficient removal of water contaminants. The PP filter has the double role of support and porous structure. The s-PBC layer provides useful additional functionalities to the commercial filter (not only size separation, but also molecules and ions removal by adsorption). This is very important since it allows to keep the filter cost low by using only a thin active coating layer on a cheap commercial PP filter. The filtration in this integrated process is assumed (understandable) as a transport and deposition complex phenomenon. To explore the possibility of further increasing the filtering efficiency of s-PBC coating, the coated filters were irradiated by UV light. The modifications induced by irradiation were investigated by FT-IR and EDX analysis.

Aiming to study the adsorption and filtration properties of the s-PBC-coated PP filters, with and without UV-induced modification, two kinds of contaminants were chosen: a cationic dye, i.e., methylene blue (MB), and two heavy metal ions, i.e., Fe^3+^ and Co^2+^, using the raw PP filter as a reference. The contaminants removal rate was measured by UV-Vis absorbance spectroscopy during both adsorption and filtration processes.

Cationic dye and metal ions were efficiently removed by s-PBC-coated PP filters due to their hydrophilic, acid and negatively charged character. In particular, the polymer-coated filters showed a high efficiency in removing MB from an aqueous solution in both absorption and filtration processes.

In the presence of heavy metal ions (Fe^3+^ and Co^2+^), on the other hand, the coated filters show a better efficiency for the filtration process, in particular in the filters whose surface has been modified by UV treatment.

The filters were characterized before and after removal processes by scanning electron microscope (SEM) energy-dispersive X-ray (EDX) analysis and FT-IR.

## 2. Results

### 2.1. Samples Characterization

Polypropylene filters were coated by s-PBC solution (s-PBC@PP), as described in the Section 3, and some of them underwent a UV irradiation process (s-PBC@PP_UV).

**Figure 1:**
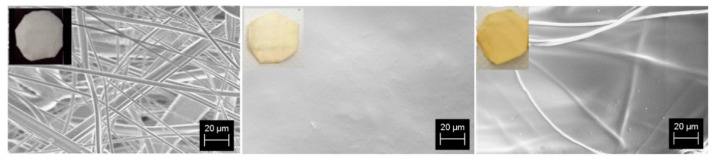
SEM images of PP (**left**), s-PBC@PP (**middle**) and s-PBC@PP after UV irradiation (**right**) samples.

Their morphology was characterized by SEM analysis, and images are reported in Figure 1. For each sample, the corresponding picture is also shown in the insets.

As we showed in previous works [19,20], the casting procedure used to cover the PP surface produces a homogeneous and complete coverage of PP fibers. Indeed, the PP filter (Figure 1, on the left) is white and is formed by a three-dimensional network of randomly distributed fibers with diameters ranging between 0.2 and 5 μm. The porosity ε_F_ is 0.98 and was determined, as described in Section 3.1 and in ref [20]. After s-PBC deposition, the sample surface turns to light yellow (Figure 1, in the middle) and the s-PBC completely covers the PP fibers as shown in the image on the right resulting in a homogeneous and smooth surface. After UV irradiation, the s-PBC layer covering PP became dark yellow (Figure 1, on the right) and the fibers become more visible under the surface, suggesting the occurrence of shrinkage or a rearrangement of the coating layer, probably due to drying, even though the sample weight does not change significantly.

The change from hydrophobic to hydrophilic behavior of the PP surface after the deposition of s-PBC and the effect of UV irradiation on s-PBC hydrophilicity was confirmed by water uptake and contact angle measurements reported in Table 1 for PP, s-PBC@PP, and s-PBC@PP_UV samples, respectively.

The starting PP surface is totally hydrophobic. The s-PBC coating results in a highly hydrophilic surface as shown by the water uptake value (262%). This value increases up to 308% for the sample after UV irradiation. The change from hydrophobic to hydrophilic behavior of the PP surface after the deposition of s-PBC was confirmed by contact angle measurements (see Figure S3 of Supplementary Materials). The average angle measured on three drops for each sample, is 129.6° for PP samples and decreases to 103.1° for s-PBC-coated PP and down to 85.6° after UV irradiation of the coating layer. In other words, the s-PBC coverage turns the PP surface character from totally hydrophobic to less hydrophobic and to hydrophilic after the UV irradiation.

The more hydrophilic character of s-PBC@PP and s-PBC@PP_UV was confirmed by FT-IR analysis in comparison with spectra acquired on PP. Previous studies [15,16] performed by FT-IR analysis on s-PBC structure demonstrate that its molecular structure in a polar solvent does not undergo chemical modification with respect to the commercial material. In fact, the IR absorption peaks characteristic of SO_3_H groups were unmodified after dispersion in polar solvent. These are responsible for the negative charge and acid character of s-PBC coating, as also confirmed by its ability to adsorb positively charged molecules [16,17,18].

**Figure 2:**
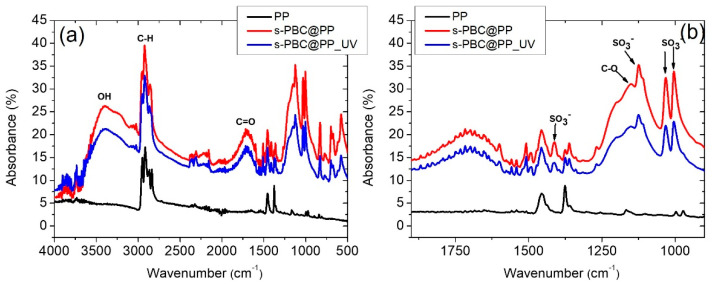
(**a**) FT-IR spectra of a PP filter (black line), s-PBC-coated PP filter before (red line) and after (blue line) the UV treatment. In (**b**), the same spectra in the wavelength range 1900–900 cm^−1^ are reported to show the IR features more in detail.

Figure 2 reports the FT-IR spectra of a s-PBC film deposited on a PP filter before (red line) and after (blue line) the UV treatment in the 4000–500 cm^−1^ wavelength range (Figure 2a) and in a reduced range between 1900 and 900 cm^−1^ (Figure 2b) to highlight some features more in detail. The IR spectrum of an unmodified PP filter (black line) is reported for comparison.

There are two “types” of signals in the IR spectrum: those related to the presence of functional groups (4000–1900 cm^−1^) and those considered to be fingerprint (1000–400 cm^−1^) typical and characteristic of the single molecules. These fingerprint signals mean that it is not possible for different molecules to have the same IR spectrum. The characteristic peaks of functional groups instead (always) fall at the same frequencies, regardless of the structure of the molecule in which the group is present.

A wide band corresponding to stretching vibrations of the hydroxyl group (OH group, from adsorbed water) occurs in the wavenumber range 3700–3000 cm^−1^ only for the filters coated with s-PBC, confirming the hydrophilicity of the polymeric coating and the totally hydrophobic behavior of the PP filter. Negligible differences in the shape of this band before and after the UV treatment were evidenced. The three peaks between 2800 and 3000 cm^−1^ are associated with asymmetric and symmetric CH- stretching vibrations from the methyl groups. The region between 2400 and 2000 cm^−1^ is strongly influenced by the presence of carbon dioxide, and the intense and broad band centered at about 1707 cm^−1^ is attributable to the C=O (carboxylic groups) stretching vibrations. Peaks evident at 1411, 1126, 1035, and 1006 cm^−1^ constitute the characteristic peaks of the SO_3_^−^ (Figure 2, right panel), whereas the peak at 1151 cm^−1^ is the C-O stretching [21,22]. The wavelength region below 900 cm^−1^ is characterized by the presence of double bonds C=C bending and C-H bending. The fingerprint area, from 1300 to 400 cm^−1^, is characterized by the presence of specific bands of each molecule, originating from vibrations of the entire molecular skeleton. The absence of new peaks after UV treatment suggests that no change of the polymer main structure occurs [23].

### 2.2. Adsorption and Filtration Tests

PP, s-PBC@PP, and s-PBC@PP_UV samples were tested for the removal of cationic contaminants, i.e., Methylene Blue, Fe^3+^, and Co^2+^ ions, both by adsorption and filtration.

Before testing the filters for contaminants removal, water flux through each filter was measured by filtering 40 mL of water in the same apparatus used for contaminants removal. The water flux for each filter, calculated as the mean value of five consecutive tests, is 18.8 mL/s for the PP filter and 4.1 and 4.9 mL/s for the ones coated by s-PBC before and after UV irradiation, respectively. The strong decrease in the water flow observed after depositing s-PBC on the PP surface is due to a reduction in the porosity of the PP filter. By comparing Figure 1a–c, we observe that the s-PBC layer completely covers PP fibers forming a compact layer, thus reducing PP porosity (also reported in [20]).

#### 2.2.1. MB Adsorption and Filtration

**Figure 3:**
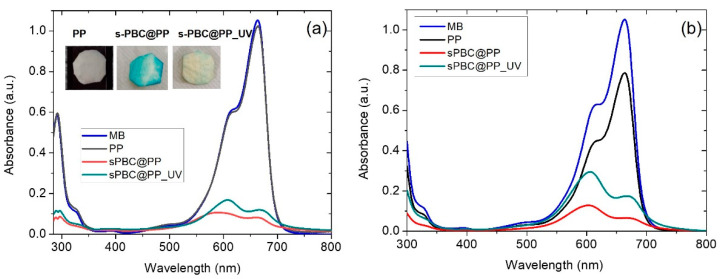
(**a**) UV-Visible absorbance spectra of MB solutions after dipping PP (black curve), s-PBC@PP (red curve), and s-PBC@PP_UV (green curve) for 3 h. In the inset a photo of each sample after three hours of adsorption; (**b**) UV-Visible absorbance spectra of MB solutions after filtration for PP (black curve), s-PBC@PP (red curve) and s-PBC@PP_UV (green curve) samples. The blue curve in both graphs represents the UV-Vis spectrum of MB at the initial concentration.

Figure 3 reports the UV-Visible absorbance spectra of MB solutions (a) after adsorption or (b) after filtration for PP (black curve), s-PBC@PP (red curve), and s-PBC@PP_UV (green curve) samples. The reference spectrum of MB solution with the initial concentration is also reported (blue curve). In the inset, a photo of each sample after three hours of adsorption is reported.

Methylene blue is a cationic, thiazine dye, which absorbs light at 664 nm (n-ð*) (monomer) with a shoulder at 610 nm corresponding to the dimer. In concentrated aqueous solutions, aggregation occurs, resulting in a shift of absorbance peak to lower wavelengths with respect to the monomer [17]. The covering of the PP surface with a layer of s-PBC makes the adsorption of MB possible due to the presence of sulfonic groups giving the surface a hydrophilic and acid character with negatively charged adsorption sites. The MB absorbance peak in solution decreased by increasing the contact time with coated samples: the main adsorption occurred suddenly, and low variations occurred in the following hours up to being constant after three hours. Thus, we report the spectra of MB solutions after three hours of contact with samples. At this time, for a solution in which s-PBC-coated PP samples were immersed, the absorbance peak of the monomer slightly shifts to higher wavelengths while the one related to dimer shifts to lower wavelengths. Their relative ratio is reduced, indicating that MB higher aggregates do not reduce linearly with monomers, or even they may form while an MB adsorption–desorption equilibrium is reached. This is not observed for the PP sample for which the adsorption is neglectable. MB removal (%) for all the samples and the amount (mg) of adsorbed MB molecules per g of deposited s-PBC layer for the coated samples are reported in Table 2 after 3 h of dipping.

The PP filter adsorbs only ~5% of MB. By covering its surface with s-PBC, a strong increase in MB removal efficiency is observed after three hours, i.e., up to 90% and 80% for s-PBC@PP and s-PBC@PP_UV, respectively. This result is also clearly visible by observing the color of samples after being immersed in MB solutions for three hours (see inset of Figure 2). The reference sample remained unaltered after being in contact with MB solution, while the s-PBC@PP sample changed its color to blue, indicating a strong MB adsorption. The UV-irradiated s-PBC@PP sample shows a removal efficiency and Qt values such as s-PBC@PP; however, its color does not turn blue after MB adsorption. This suggests that MB is converted into solution or adsorbed on the sample surface in its colorless form (i.e., leuco) as a result of a reduction process of MB molecules; this reduction could be ascribed to a modification induced on the sample surface by UV irradiation that enriches its surface of electrons. When MB is reduced to its leuco form, the main absorbance peak of MB at 664 nm disappears, and a new absorbance peak in the UV range appears, i.e., 254 nm [24]. This peak is not observed in the absorbance spectra of the residual solution, thus suggesting that MB is reduced to its leuco form only after adsorption on the s-PBC@PP_UV sample.

The leuco MB species is not observed when MB is removed by filtration using PP, s-PBC@PP, and s-PBC@PP_UV filters. Indeed, the s-PBC@PP filters treated or not by UV irradiation became blue after filtration while the eluate was uncolored. This could be ascribed to the processing time: filtration occurred in less than one minute while adsorption occurred in three hours. In the last case, the contaminant molecules have more time to interact with radical species generated within the polymeric matrix during its irradiation [25].

PP alone (black curve) removes about 20% of MB after the first filtration process, and then this percentage decreases with subsequent filtrations, possibly due to a release of MB from the filter (spectra not shown here). For the s-PBC@PP (red curve) sample, the filtration removal efficiency reaches 80% for the first process, and for the s-PBC@PP_UV (green curve) sample, the removal is lower (about 70%). In both cases, the peak associated with dimers and oligomers at about 600 nm is more intense than the one due to the monomer at 664 nm.

Table 2 reports the Q_t_ values also for filtration processes: s-PBC@PP shows a Q_t_ value of 0.45 mg/g that reduces to 0.38 mg/g after UV irradiation of the s-PBC layer. These values are lower but quite similar to the one reported for adsorption tests suggesting that a longer contact time between filter and contaminants (3 h for adsorption process vs. 1 min for filtration) results in better removal efficiency. However, in terms of time required for removal, filtration is clearly more advantageous and can be repeated consecutively to improve the final result.

#### 2.2.2. Fe^3+^ Adsorption and Filtration

**Figure 4:**
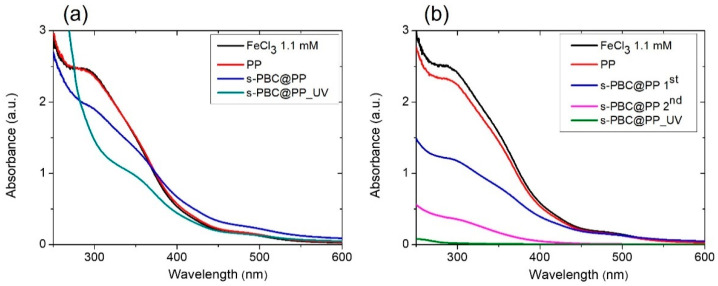
UV-Visible absorbance spectra of FeCl_3_ solutions after (**a**) adsorption on the different filters or (**b**) filtration through them. The reference spectrum of the initial FeCl_3_ solution is reported in both graphs.

Figure 4 reports the UV-Visible absorbance spectra of FeCl_3_ solutions where filters were immersed in the dark for three hours (Figure 4a) or after filtration (Figure 4b). The red curve represents the PP filter whose adsorption is neglectable, and only a small decrease in Fe^3+^ concentration is observed after filtration (i.e., 20% Fe^3+^ reduction). After covering the filter with s-PBC, the adsorption ability increased up to 75% with respect to PP itself (see the blue curve of Figure 4a), and this is due to the hydrophilicity and negative charge of the s-PBC layer surface. A similar result was obtained for filtration using the s-PBC@PP filter: the Fe^3+^ concentration was reduced by 50% at the first filtration (see the blue curve of Figure 4b) and up to 80% by filtering the same solution for the second time through the same filter (see the magenta curve of Figure 4b). The UV irradiation of s-PBC layers worsened the adsorption ability (i.e., 28% Fe^3+^ reduction, see the green curve of Figure 4a) but highly increased the filtration efficiency since the total removal of Fe^3+^ ions was observed immediately after the first filtration process (see the green curve of Figure 4b), due to the higher hydrophilicity of the sample.

However, observing the shape of the spectra after absorption and filtration of the solution, they show a different shape for the sample treated with UV. In fact, for the s-PBC@PP filter, the spectra of the absorbed and filtered solutions are similar in shape, both with each other and with the initial solution. The only difference is represented by the intensity, which is lower for the filtered solution, indicating a release of Fe^3+^ ions from the sample during the three hours of absorption. For the s-PBC@PP_UV filter, the spectra of the absorbed and filtered solution show different shapes, both with each other and with the initial solution. In particular, while the spectrum after filtration shows only a strong variation in intensity (>99% of the ions were removed), the spectrum after adsorption shows the appearance of a very intense absorbance signal for wavelengths lower than 300 nm. From the literature [26], it is known that Fe ions show absorption peaks whose position varies according to the oxidation number (Fe^2+^ and Fe^3+^) and the group to which the Fe ion is bound (Fe(OH)^2+^, Fe(SO_4_)_2_^−^, FeSO_4_^+^, FeCl_2_^+^, FeCl^2+^, …). So, considering that the shape of the UV-Vis spectrum changed for the s-PBC@PP_UV filter after three hours of contact with the iron solution in the dark, we believe that the change is due to the formation of different iron species in the solution. Accordingly, the UV-irradiated layer reacts with iron ions in solution instead of adsorbing them.

The s-PBC coating of the PP filter increased its Fe^3+^ removal efficiency with respect to PP by both adsorption and filtration. Higher adsorption efficiency is shown by the s-PBC@PP filter (i.e., 7.75 mg/g), while UV irradiation reduces this performance (i.e., 2.8 mg/g). On the contrary, the UV-irradiated filter showed a higher removal efficiency by the first filtration test (up to 8.48 mg/g). A similar result was shown by the s-PBC@PP filter after filtering twice the same solution through the same filter (i.e., 7.96 mg/g).

#### 2.2.3. Co^2+^ Adsorption and Filtration

**Figure 5:**
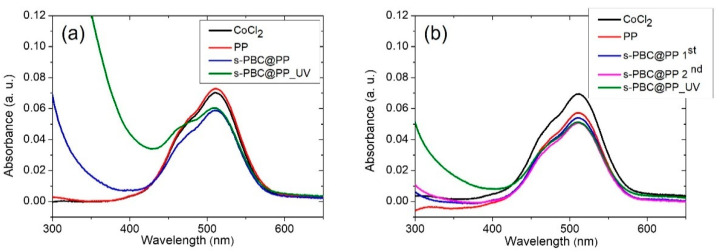
UV-Visible absorbance spectra of CoCl_2_ solutions after (**a**) adsorption on the different filters for two hours or (**b**) filtration through them. The reference spectrum of the initial CoCl_2_ solution is reported in both graphs.

Figure 5 reports the UV-Visible spectra of (i) as-prepared CoCl_2_ solution and the same after contact with different filters in the dark for three hours (Figure 5a); (ii) as-prepared CoCl_2_ solution and the same after filtration (Figure 5b). The main peak associated with Co^2+^ is positioned at 520 nm. The red curve represents the initial PP filter whose adsorption is neglectable. After coating the filter with s-PBC, the adsorption ability increases, and 15% of the initial Co^2+^ concentration is removed (see the blue curve of Figure 5a) due to the more negatively charged surface of the s-PBC layer with respect to the totally hydrophobic PP surface. For the UV-irradiated s-PBC layers, 14% Co^2+^ reduction is observed (see the green curve of Figure 5a), indicating that the UV treatment did not significantly affect the adsorption ability with respect to the s-PBC@PP filter. However, similarly to the case of Fe^3+^ ions described in the previous section, a significant variation in the shape of the absorbance spectra occurs at wavelengths below 400 nm after the prolonged interaction of the filters with the Co^2+^ ions solution.

After filtration through the PP filter, Co^2+^ concentration decreases by 15% (red curve in Figure 5b). A slightly better result was obtained using the s-PBC@PP filter: 20% of the initial Co^2+^ concentration was removed after the first process (see the blue curve of Figure 5b) and 28% by re-filtering the same solution twice through the same filter (see the magenta curve of Figure 5b). The same decrease (28%) of the 520 nm peak was observed for the s-PBC@PP_UV filter, along with a moderate absorbance increase at low wavelengths (below 400 nm).

The higher removal efficiency observed for filtration may be mainly ascribed to the higher hydrophilicity of polymer-coated filters after UV irradiation.

We believe that the large increase in the absorbance at low wavelength values observed for s-PBC@PP_UV, more evident in the case of adsorption but also present after filtration, might be due to the formation of different cobalt species in solution as byproducts of a reaction occurring between the UV-irradiated polymer layer and the cobalt ions in solution. This could explain the absorbance increase observed in the UV range of the spectrum and the very small decrease in the 520 nm peak.

The Q_t_ values for cobalt removal by adsorption and filtration were calculated according to the definition given in the Section 3. Coating the PP filter by an s-PBC layer allows a moderate Co^2+^ removal by adsorption, i.e., Q_t_ = 21.9 mg/g (no adsorption is observed for raw PP filter) that slightly increases (Q_t_ = 24.0 mg/g) by filtration. UV irradiation lowers the removal efficiency of the filter to 20.0 mg/g and 17.3 mg/g for adsorption and filtration, respectively.

To understand in more detail these results, we have performed FT-IR and EDX analysis on the three kinds of filters after adsorption and filtration of Fe^2+^ and Co^2+^ ions.

### 2.3. Post-Process Filters Characterization

#### 2.3.1. FT-IR Analysis

**Figure 6:**
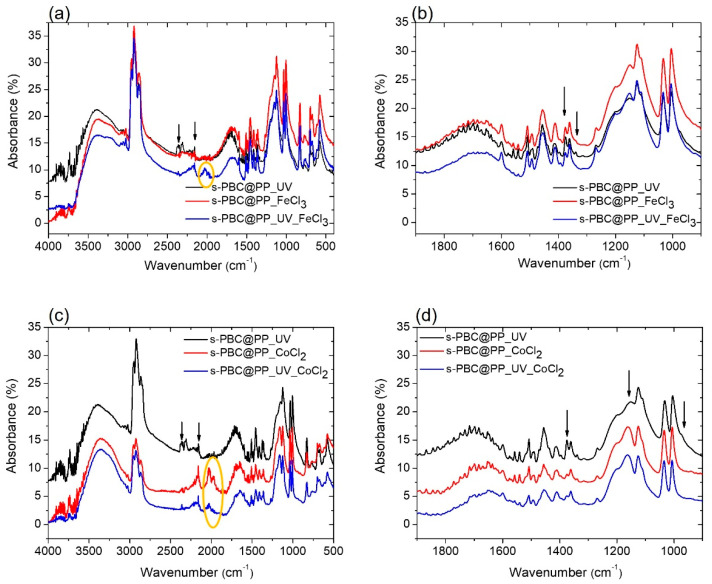
FT-IR spectra of (**a**,**b**) s-PBC@PP filter (red line) and UV-treated s-PBC@PP filter (blue line) after being dipped in a FeCl_3_ solution; (**c**,**d**) s-PBC@PP filter (red line) and UV-treated s-PBC@PP filter (blue line) after being dipped in a CoCl_2_ solution. The (**b**,**d**) figures show more in detail the 1900–900 cm^−1^ range. FT-IR spectra of the s-PBC@PP filter after UV treatment is reported in all the graphs as a reference (black line).

Figure 6 shows the FT-IR spectra of an s-PBC@PP filter after UV treatment (black line) and the s-PBC@PP filters without and with UV treatment after being dipped in a 1.1 mM FeCl_3_ solution (red and blue line, respectively, Figure 6a,b) or after being dipped in a 17.5 mM CoCl_2_ solution (red and blue line, respectively, Figure 6c,d). As a reference, we report the s-PBC@PP after UV treatment because it shows the same features as the untreated s-PBC@PP, as shown in Figure 3.

After the adsorption of Fe^3+^, the main differences observed among the spectra are in the ranges 1900–2350 cm^−1^ and 1400–1100 cm^−1^: we observe the presence of two large features in the range 2250–1900 cm^−1^ only in the case of the UV-treated s-PBC@PP sample. The peaks centered at approximately 2350 and 2157 cm^−1^ (indicated by the arrows in Figure 6a) are present in all the filters and are related to the O=C=O and C=C=O bonds, respectively. The peaks centered at 2027 e 1973 cm^−1^ and circled in yellow have not been identified. Further differences concern the band centered at 1707 cm^−1^ (related to the C=O stretching vibrations) that shifts to 1679 cm^−1^, and the decrease in relative intensities of the peaks at 1375 cm^−1^ (S=O), 1338 (S=O) cm^−1^ indicated by the arrows in Figure 6b.

In the case of Co^2+^ adsorption, the two features in the range 2250–1900 cm^−1^ are present for both s-PBC@PP with and without UV treatment, and, in particular, the peaks centered at 2027 e 1973 cm^−1^ and circled in yellow are present (Figure 6c), more evident for the untreated sample. The band centered at 1707 cm^−1^ (related to the C=O stretching vibrations) shifts to 1650 cm^−1^, and the intensity of the peaks at 1375 cm^−1^ and 1338 cm^−1^ (both related to S=O stretching) decrease, such as in the case of Fe^3+^ adsorption. Furthermore, the 1153 cm^−1^ peak (related to C-O stretching) shifts to 1161 cm^−1^, and its relative intensity increases after the interaction with CoCl_2_, as indicated by the arrows in Figure 6d.

The results of our adsorption/filtration tests indicate that after the contact between s-PBC@PP and s-PBC@PP_UV samples with FeCl_3_ and CoCl_2_ solutions: (i) the amount of S=O bonds decreases; (ii) the peaks related to C=O (1707 cm^−1^) and C-O (1153 cm^−1^) stretching shift, respectively, to a lower and to a higher value, suggesting that there is a change in the electron distribution of the molecular bonds, probably due to strong interaction with cationic ions. All the observed changes (change of relative intensity and shifts) are much more evident in the case of contact with the CoCl_2_ solution, highlighting a stronger interaction of sulfonilic groups with Co^2+^ with respect to Fe^3+^ ions.

#### 2.3.2. EDX Microanalysis

**Figure 7:**
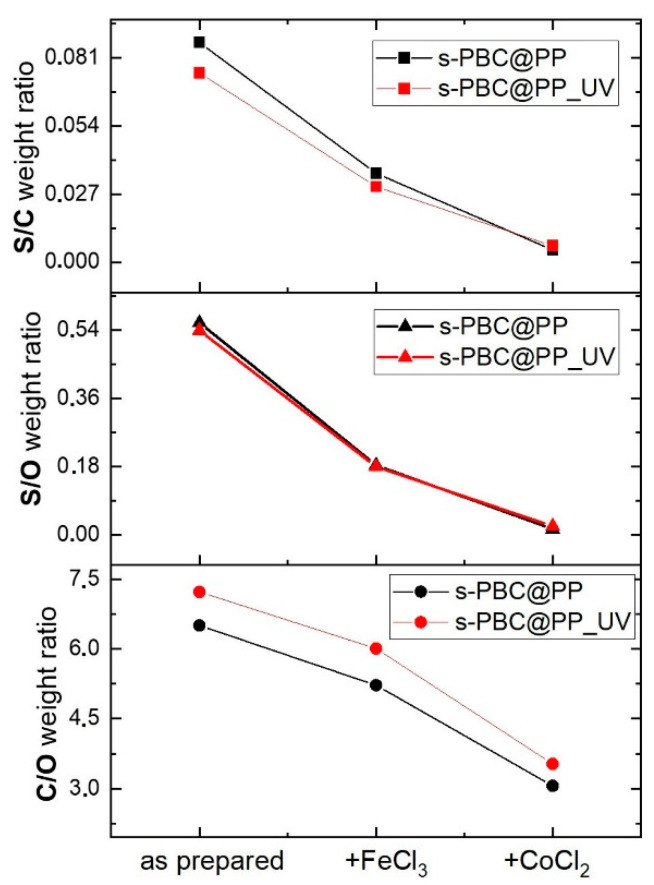
C/O, S/O, and S/C weight ratios measured by EDX analysis for s-PBC@PP and s-PBC@PP_UV samples before and after dipping in FeCl_3_ solution or in CoCl_2_ solution for 180 min.

Figure 7 reports the C/O, S/O, and S/C weight ratios obtained by EDX analysis carried out on s-PBC@PP and s-PBC@PP_UV samples before and after being dipped for 180 min in a 1.1 mM FeCl_3_ solution or in a 17.5 mM CoCl_2_ solution. The comparison between the s-PBC layer before and after UV irradiation (black and red curves, respectively) indicates that the UV treatment slightly changes the relative amounts of O and S with respect to C. In particular, we observe a decrease in the S/C ratio and an increase in the C/O ratio, while S/O remains almost unvaried.

The S/C and S/O weight ratios measured for the samples exposed to FeCl_3_ and CoCl_2_ strongly decrease with respect to the values measured for the corresponding as-prepared samples, and this effect is enhanced in the case of immersion in the CoCl_2_ solution. The C/O weight ratio decreases moderately for the samples in contact with the FeCl_3_ solution and much stronger for the samples in contact with the CoCl_2_ solution. No significant difference in the general trend is observed between s-PBC@PP and s-PBC@PP treated by UV irradiation.

A decrease in the S content in the s-PBC coating is in agreement with the FT-IR results, where a reduction in the S=O stretching features (1376 cm^−1^, 1338 cm^−1^) is observed. EDX provides the additional information that this decrease is strongly enhanced by the presence of CoCl_2_ with respect to FeCl_3_.

An increase in O content seems to be supported by smaller C/O and S/O ratios after contact with both solutions and by the increase in the IR peak at 1153 cm^−1^ assigned to C-O stretching. Changes in the electron distribution of the molecular bonds between C and O are also suggested by the downshifts observed in the FT-IR features from 1707 cm^−1^ to 1679–1650 cm^−1^ (C=O stretching) and by the upshift from 1153 to 1161 cm^−1^ (C-O).

These results suggest that adsorption/filtration of ions, in particular, cobalt, occurred by coordination with sulfonilic groups that dissolve in solution as sulfate salts, as demonstrated by the presence of byproducts shown in Figure 4 and Figure 5.

## 3. Materials and Methods

### 3.1. Chemicals

Multilayer polypropylene (PP) filters were produced using the melt-blown technology process, as reported in [19,27].

The produced PP fibers have diameters smaller than 5 mm and form a three-dimensional network with a range distribution of the pore size. The filter porosity ε_F_ is 0.98 and was determined using the formula: ε_F_ = 1 − ρ_SF_/(ρ_F_·L), where r_SF_ is the surface density calculated as the mass of the filter divided by the surface area (m_F_/A_F_); ρ_F_ is the fibers material density (910 Kg/m^3^ for polypropylene was used); L is the PP filter thickness.

A sulfonated pentablock copolymer poly(tBS–HI–sS:S–HI–tBS) solution, or s-PBC, with 10–12 wt% polymer in a cyclohexane/heptane mixed solvent was provided courtesy of Kraton Polymers LLC. A scheme of this copolymer, commercially available as Nexar^TM^, is reported in Figure S1 of Supplementary Materials [15,16,17,18]. The sulfonation degree is 52 mol%, and these groups confer to the polymer an ion exchange capacity (IEC) value of 2.0 meq/g. The molecular weight is 112,500 g mol^−1^ and the volume fraction (tBS–[sS:S]–HI) is 0.300–[0.226:0.208]–0.266 [28]. The structure of this polymer is composed of an alternation of hydrophobic and hydrophilic domains within a micellar morphology in solution, depending on the solvent polarity [28]. This morphology affects the morphology of casted film: discrete, spherical ion-rich microdomains are formed in films cast from nonpolar solvent, whereas an apparently mixed morphology with a continuous ion-rich pathway and channels is generated when the casting solvent is more highly polar [28]. The presence of these pathways and channels could facilitate the diffusion of ions and/or other polar species through the nanostructured medium. Taking into consideration these observations, we prepared an s-PBC solution (3 wt%) by dispersing the commercial s-PBC after evaporation of commercial solvents in a polar solvent, i.e., isopropyl alcohol (IPA), since the obtained structure could be more suitable for filtration purpose.

### 3.2. Samples Preparation

For adsorption tests, polypropylene filters were cut into circular coupons of ~0.3 cm thickness and ~2.25 cm diameter. A 1 mL volume of s-PBC solution was spotted on the PP coupon to obtain a homogeneous coverage of its surface. Each coupon was air-dried for 24 h before weighing. The amount of deposited s-PBC was ≈0.030 ± 0.01 g for each coupon. Similarly, we prepared larger coated filters for filtration processes: polypropylene filters were cut into circular coupons of ~0.3 cm thickness and ~5 cm in diameter. These were coated with 5 mL of s-PBC solution, and each filter was air-dried for 24 h before weighing. The amount of deposited s-PBC was ≈0.1468 ± 0.0030 g for each filter as measured by a precision weight scale. PP samples covered by s-PBC are here named s-PBC@PP samples.

Some s-PBC@PP samples underwent a UV irradiation process for 7 h, respectively (here named s-PBC@PP_UV). The irradiation was performed by an 18 W UVA/blue DULUX n.78 OSRAM lamp (producing mainly UV emission at 365 nm and a few narrow lines in the visible). The emission spectrum of the lamp is reported in Figure S2 of Supplementary Materials.

The irradiated and unirradiated s-PBC@PP samples were characterized and tested for water contaminants removal as described below. These were compared with unmodified PP samples.

### 3.3. Samples Characterization

Morphological characterization and chemical mapping of the samples were performed using a field emission scanning electron microscope (Supra 35 FE-SEM by Zeiss, Oberkochen, Germany) equipped with an energy-dispersive X-ray (EDX) microanalysis system (X-MAX, 80 mm^2^ by Oxford Instruments, Abingdon, UK).

The hydrophilicity of the sample’s surface was investigated by contact angle measurements using an Optical compact angle meter (CAM 200 model by KSV Instruments LTD, Helsinki, Finland). Moreover, their water uptake values were calculated according to the following simple mass balance equation [15,16,17,18]:

Uptake% = [(m_wet_ − m_dry_)/m_dry_] × 100 (1)

where m_dry_ is the mass of the sample air-dried at least for 24 h and then put into a desiccator; m_wet_ is the weight of the sample soaked in distilled water at room temperature for 48 h and quickly wiped with a paper tissue in order to remove most of the free surface water. Both masses were measured by a microbalance.

IR measurements on irradiated and not covered PP filters were performed by Jasco FT-IR-4700 spectrophotometer. Equipped with an ATR (ATR-PRO ONE) with a diamond prism. Clamps ensure sample contact with the crystal.

Water flux through the initial PP filter and s-PBC-coated filters before and after UV irradiation, respectively, was measured by the ratio of water volume on filtration time for five consecutive filtration processes of a 40 mL water sample using a vacuum filtration unit with a diaphragm vacuum pump. The same system was used for filtration experiments, as reported below.

### 3.4. Adsorption and Filtration Tests

s-PBC@PP and s-PBC_UV samples were tested for the adsorption and filtration of a cationic molecule (i.e., methylene blue MB) and cationic metals such as Fe^3+^ and Co^2+^ from water. For adsorption experiments, samples were immersed in the dark in 5 mL of contaminant aqueous solutions with an initial concentration of 10^−5^ M for MB, 1.1 mM for FeCl_3_, and 17.5 mM for CoCl_2_, respectively, for three hours. For each filtration process, three different 20 mL aliquots of initial contaminant solution were filtered, and each eluted aliquot was filtered for consecutive cycles. The filtration tests were conducted through commercial vacuum filtration systems (Nalgene^®^). In the systems, the membrane in PES Supor^®^ machV was removed and replaced with the filters to be tested. The mean flow (mL/s) was calculated for all filters by filtering 40 mL of H_2_O 4 times. The mean values obtained were 4.18, 4.11, and 4.87 mL/s for PP, s-PBC@PP, and s-PB@/PP_UV, respectively. Filtration time was recorded for each process. All the solutions before and after adsorption and filtration were analyzed by recording the absorbance spectra variations using a UV/Vis AGILENT Cary 50 spectrophotometer in a wavelength range between 200 and 800 nm. The contaminant removal was evaluated by the Lambert–Beer law via the absorbance peak at 664 nm for MB, 286 nm for Fe^3+^, and 520 nm for Co^2+^. Adsorption and filtration processes on PP filters used as references were also evaluated to point out the role acted by s-PBC covering.

The efficiency of adsorption and filtration tests is measured as an amount (%) of removed ions or as a weight ratio (Q_t_, mg/g) between removed ions and the s-PBC amount of the coating layer.

## 4. Conclusions

New technical solutions for functionalizing materials by assigning them defined structural features create the possibility of integrating processes to increase the separation efficiency and reduce the operational costs of the process. This paper describes an example of such an operation for the case of water purification using coupled adsorption and filtration processes.

In this work, we coated PP filters with s-PBC layers and tested them for the removal of cationic organic dyes, such as methylene blue (MB), and heavy metal ions (Fe^3+^ and Co^2+^) from water by adsorption and filtration experiments. Some of the coated filters were irradiated by UV light before being exposed to contaminated water and then were tested with the unirradiated filters in the same adsorption and filtration experiments.

S-PBC coating made PP filters partially or totally able to remove the cationic dye and metal ions from water solutions due to their hydrophilic, acid, and negatively charged character.

In particular, the polymer-coated filters showed high efficiency in removing MB from an aqueous solution in both adsorption and filtration processes, with efficiencies of 90% and 80%, respectively.

In the presence of heavy metal ions (Fe^3+^ and Co^2+^), on the other hand, the coated filters showed a better performance for the filtration process rather than in the adsorption processes. In particular, the UV treatment improves the removal ability of Co^2+^ ions (about 28% rather than 20% obtained for the untreated s-PBC@PP filters) and allows a total removal of the Fe^3+^ ions (>99% instead of 50% achieved by the untreated s-PBC@PP filters) after just one single filtration step.

The characterization of the filters before and after use by FT-IR and EDX analysis allowed us to investigate the mechanisms involved in the metal ions removal processes: in the adsorption processes, the interaction times are long enough to allow the ionic species to interact with the surface of the filters, leading to the formation and release of new species in solution. The filtration processes are faster, and, therefore, the interaction time is not enough to release reaction byproducts to the solution; the ionic species are easily trapped in the filters, in particular for the filters whose surface was modified by UV treatment. It has also been shown that the treatment increases the hydrophilicity of the filters, enhancing their filtration capacity. Although further work is needed to extensively investigate the lifetime and regeneration processes of such kinds of filters, we have shown that functional polymeric coating of commercial and low-cost filters is a promising and low-cost strategy for the effective removal of pollutants from water.

## Figures and Tables

**Table 1:** Water uptake (%) and contact angle measured for PP, s-PBC@PP, and s-PBC@PP_UV samples.

Sample	Water Uptake (%)	Average Contact Angle (Deg)
PP	3	129.6
s-PBC@PP	262	103.1
s-PBC@PP_UV	308	85.6

**Table 2:** MB removal (%) and amount of adsorbed dye per gram of adsorbent (Q_t_, mg/g), considering the peak at 664 nm after adsorption and filtration tests.

Sample	MB Removal by Adsorption (%)	Q_t_ads_ (mg/g)	MB Removal by Filtration (%)	Q_t_filt_ (mg/g)
PP	5	-	20	-
s-PBC@PP	90	0.58	80	0.45
s-PBC@PP-UV	80	0.56	70	0.38

## Data Availability

The data presented in this study are available on request from the corresponding authors.

## Supplementary Materials

The following supporting information can be downloaded at: https://www.mdpi.com/article/10.3390/ijms231911777/s1.
