# Supplementary material for: Sulfonated Pentablock Copolymer Coating of Polypropylene Filters for Dye and Metal Ions Effective Removal by Integrated Adsorption and Filtration Process
Source: Int J Mol Sci. 2022 Oct 4;23(19):11777. doi: 10.3390/ijms231911777 (PMC9570310; doi:10.3390/ijms231911777)
Supplement: Supplementary file 1 [file ijms-23-11777-s001.zip › ijms-1916108-supplementary.pdf]

International Journal of
*Molecular Sciences*

*Supplementary Materials*

# Sulfonated Pentablock Copolymer Coating of Polypropylene Filters for Dye and Metal Ions Effective Removal by Integrated Adsorption and Filtration Process

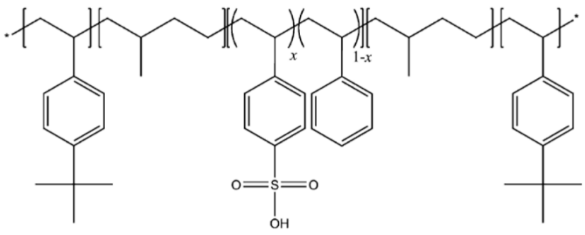


**Figure S1.** Molecular structure of sulfonated pentablock copolymer (s-PBC) (Nexar™).

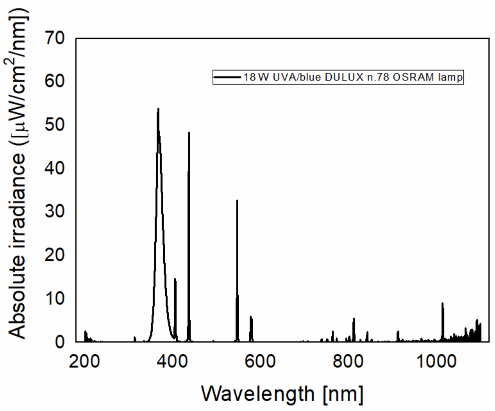


**Figure S2.** The emission spectrum (UV emission at 365 nm and a few narrow lines in the visible) of the 18 W UVA/blue DULUX n.78 OSRAM lamp used for irradiation of the coating layer.

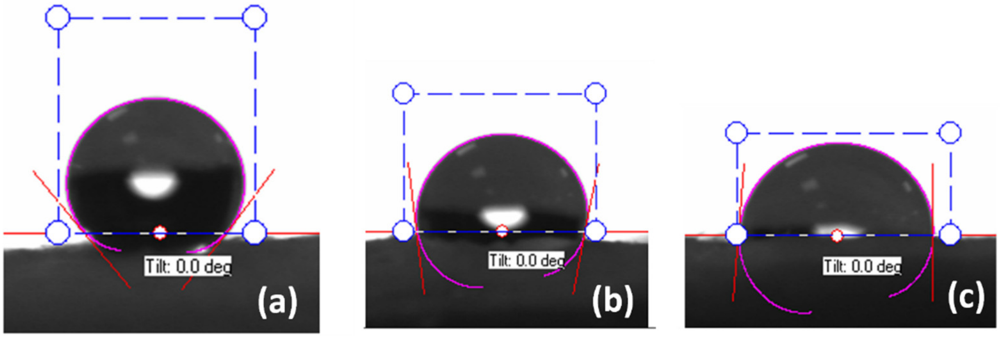


| Sample | Average value (left side) | Average value (right side) |
|---|---|---|
| PP | 130.8 | 128.4 |
| S-PBC@PP | 102.4 | 103.9 |
| S-PBC@PP_UV | 84.4 | 86.8 |

**Figure S3.** Contact angle measurements for different filters: (**a**) PP, (**b**) s-PBC@PP (center) and(**c**) s-PBC@PP after UV irradiation. The table reports the average values measured on the left and right side of three different drops for each sample.
